# Supplementary material for: Are carbon emissions trading and green financial instruments synergistic? -Comprehensive quantitative research based on content analysis
Source: PLoS One. 2024 Mar 7;19(3):e0298601. doi: 10.1371/journal.pone.0298601 (PMC10919601; doi:10.1371/journal.pone.0298601)
Supplement: S1 Data — (ZIP) [file pone.0298601.s001.zip › Data/地级市Z_F工作报告.zip!/┴╔─■/╔≥╤⌠╩╨/└┤╫╘╣1⁄2╓┌║┼í╛┬φ┐╦╩2╛▌═°í┐_2003.docx]

　　现在，我代表市政府向大会作工作报告，请予审议，并请市政协委员提出意见。

　　一、过去五年的工作回顾

　　过去的五年，在省委、省政府和市委的正确领导下，在市人大、市政协的支持和监督下，市政府带领全市人民高举邓小平理论伟大旗帜，努力实践“三个代表”重要思想，实现了国民经济和各项社会事业的快速发展。

　　———经济总量持续增长，综合经济实力显著增强。预计2002年全市国内生产总值实现1400亿元，同比增长13.1％，比1997年增长67.6％，是九十年代中期以来增长最快的年份；固定资产投资完成402亿元，同比增长32.8％，是1997年的2.41倍；财政收入实现105亿元，同比增长29.9％，连续两年保持了30％左右的增长，是1997年的2.19倍；城市居民人均可支配收入达到7050元，农民人均纯收入达到3500元，同比分别增长10.4％和8.4％，比1997年分别增长49.6％和45.9％。五年来有56万贫困人口解决了温饱问题。

　　———农业结构调整全面铺开，农村经济取得新发展。全市农村二、三产业比重达到87.3％，粮经作物比例稳定在6：4,畜牧、水产业占农业的比重达到37.7％。去年郊区、县（市）预计实现财政收入12.7亿元，同比增长21％，是1997年的2.1倍。

　　大规模开展了退耕还林和退耕还草，去年全市完成植树造林55.3万亩，新开发草原8万亩。农产品出口基地、现代农业园区和农业科技示范园建设取得新进展，沈阳农业高新区建设全面启动。乡镇企业发展取得长足进步。

　　去年市政府投资6000万元，带动县区城建投资3.5亿元，促进了县城和副城建设。

　　———拓展新的发展空间，为全面振兴工业奠定基础。2001年，全面启动了浑南新区的开发建设。目前，全区120平方公里的总体规划已经完成，“三区一城一带”已见雏形；光科通讯等27个高科技项目已经落户新区，863软件园等8个高新技术产业园建设全部启动，开工建设了道路、桥梁等40余个基础设施项目。

　　为了促进先进装备制造基地建设，将铁西区和沈阳经济技术开发区领导班子合署办公，并积极推进两区的合并。目前新区的政策、体制效应已初步显现，大调整、大发展的格局开始形成。老工业区面貌明显改观。去年，铁西新区共引进项目131个，搬迁企业50户。

　　2001年以来，在绝大多数县区设立了工业园区。目前，这些园区已经成为县区招商引资的重要载体。

　　——国有经济的战略性调整取得了新突破，民营经济发展开创了新局面。在完成国有企业三年改革脱困任务的基础上，全市国有企业改革进一步深化。两年来，财政支付改革成本2.7亿元，完成了52户国有大中型企业的主辅分离工作；推出20户大型企业进行整体合资，现已完成5户；完成了516户国有中小工业企业的转属工作，其中80％完成转制；对142户资不抵债企业实施了破产；财政直接支付4.45亿元资金，对19万名国有企业下岗职工实施了向失业保险并轨。

　　在全市国有企业改革的历史上，近两年是减轻企业负担最多、企业得到实惠最大的两年。由于采取了强有力的措施，一大批国有企业在困境中崛起，重新焕发了活力。去年，预计全市国有及国有控股工业企业实现产值同比增长19.5％，为近年来少见。

　　我们还通过引进外地民营企业，扶持本地民营企业，推进国有中小企业转制，形成了三路大军共同发展民营经济的新局面。去年全市民营经济实现增加值741.6亿元，占全市国内生产总值的52.9％，比1997年增长1.26倍。

　　——服务贸易业迅猛发展，区域中心城市地位进一步巩固。大力引进连锁经营、现代超市、物流配送等新型商业业态，积极发展金融、旅游、房地产、信息等现代服务业，不断完善人才、技术、土地、产权交易等要素市场体系，成功举办一批大型展洽活动，使沈阳的中心城市地位和服务功能不断提升。去年，全市社会消费品零售总额预计实现695亿元，比1997年增长63.7％；各类市场成交额达到812亿元，比1997年增长52.3％；金融机构存、贷款余额分别达到2190亿元和1820亿元，比1997年分别增长了102％和87％；货币净回笼连续12年居全国之首。

　　我们以应对入世挑战，创造中国北方最佳投资环境为目标，制定并落实了改善投资环境的十五项重点工作共50条具体措施。将原有的1229项审批事项，削减至284项，并全部进入在市区设立的七个审批办事大厅。同时，我们还加大了对国内外投资者历史遗留问题的处理力度，使沈阳市的投资环境明显改善。

　　——努力扩大对外开放，招商引资工作开创了新局面。2001年实际利用外资取得突破，达到12.08亿美元，同比增长15.7％，其中外商直接投资8.5亿美元；2002年再创新高，预计实现16.5亿美元，增长36.8％，其中外商直接投资14亿美元，同比增长64.6％。这样的增长在全国也处于先进行列。全市进出口总额预计实现29亿美元，同比增长4.7％，其中出口预计实现14亿美元，增长11.9％。现在，全市税收的三分之一、出口创汇的三分之二、固定资产投入的四分之一以及技术改造投资的三分之一，均来自外商投资企业。

　　对外交流和友好往来不断扩大。包括加纳总统在内的众多高层次外宾来沈访问；我市与11个国家的12个城市建立了友好城市关系；新开通3条国际航线，使沈阳的国际航线达到了14条。去年我们成功举办了“国际装备制造业博览会”，提高了我市的国际知名度，扩大了对外影响力。

　　——以提高城市品位和贴近市民生活为宗旨，大力推进城市建设。两年来，全市城建累计投资89.3亿元，是前三年总和的2.14倍。

　　为了改变沈阳缺水少绿的局面，创建独特的城市品牌，我们提出了建设森林城市的奋斗目标。去年在人代会通过绿化年活动方案后，全市开展了声势浩大的绿化年活动，新增绿化面积17.7平方公里，相当于已有绿化总面积的33.9％，城市绿化覆盖率跃升到29.1％。

　　完成了市府大路等10条街路的标准化改造，对213条市区道路进行了全面整修，总里程达到265公里，总面积751万平方米，使全市道路完好率由过去的70％提高到90％。

　　浑河整治取得了重大突破，城市段水质明显改善。浑河成为城市内河，使沈阳的城市格局发生了质的变化。去年，通过拆除1125根烟囱及400座锅炉房，推广使用型煤，治理汽车尾气，使大气优良天数从2001年的73天，提高到2002年的170天。

　　绿树、碧水、蓝天不再是沈阳人的梦想，正在成为我们生活中的现实。

　　两年来，5.7万户棚户区居民迁入新居；解决了11.6万户居民煤气拖期开栓和57万人“三峰”供水的问题；新增公交车1250辆；实施了新的除运雪办法，供暖质量明显好于往年。土地出让金收入由2000年的7000万元跃升到2001年的10亿元，去年又达到15亿元。土地出让金已成为城市建设资金的主要来源。

　　——扎实做好试点工作，社会保障体系日益完善。目前，全市有118.7万人参加企业养老保险并实现了养老金100％的社会化发放，133万人参加基本医疗保险，130.1万人参加失业保险，17.9万人享受最低生活保障，实现了应保尽保。社保体系的建立和不断完善，对沈阳来说意义非同寻常，其难度也可想而知。坚持实施再就业工程，仅去年就开发就业岗位12万个，实现再就业18.3万人次。

　　——努力建设大文化，社会事业不断发展。去年，全市高新技术产品产值预计实现620亿元，是1997年的2.9倍，占全市工业总产值的29.4％。全面启动了利用高新技术改造传统装备制造业的11个科技专项，其中6个项目列入国家计划。启动了人才资源高地计划，引进国外专家432名。

　　教育综合改革进一步深化，全面完成“普九”达标任务，高校体制改革取得明显进展。适龄儿童入学率达到99.8％，初中入学率达到99.1％，农村初中辍学率从1997年的6.5％下降到2002年的3.64％。

　　文化事业日益繁荣，《天幻》等一批优秀剧目在国内外获得大奖，群众文化活动广泛开展。城镇医疗卫生体制改革全面展开，农村卫生工作得到进一步加强。体育事业有了长足进步，全民健身运动蓬勃开展。前年，我们成功地承办了世界杯足球亚洲区十强赛，提升了广大市民的自信心，向全世界展示了沈阳的良好形象。

　　精神文明创建活动扎实推进，市民素质和文明程度进一步提高。民主法制建设不断加强，依法治市工作取得了新进展，五年来，制定政府规章71件。去年按照入世要求废止政府规章272件，有力地促进了依法行政。加强了基层政权建设，社区建设取得了新进展。全面完成了机构改革任务。政府职能和机关作风切实转变，党风廉政建设大力加强。各级政府自觉接受人大、政协和人民群众的监督，去年办理建议和提案1730件，办复率达到100％。

　　有效地打击了“法轮功”邪教组织。深入开展以“打黑除恶”、“扫黄打非”为重点的严打整治工作，社会治安明显好转。努力化解社会热点问题，社会稳定得到良好保障。军民、军政团结不断加强，军民共建取得新进展。新闻、出版、广播、电视、民族、宗教、信访、统计、审计、物价、安全生产、技术监督、计划生育、民兵、预备役、人防、档案、修志、文史、参事、残联和老龄工作也取得了新的成果。

　　各位代表，过去的五年，是全市经济不断发展，社会事业不断进步的五年，是城市面貌不断改变的五年，也是全市人民生活水平不断提高的五年。在这期间，发生了“慕、马”案件，我们在市委统一领导下，积极开展了反腐败斗争，克服了“慕、马”大案带来的严重影响，排除各种干扰，战胜了重重困难，经受了严峻考验，重塑了政府形象，依靠全市广大干部群众自强不息的奋斗，使沈阳的各项事业不仅没有出现倒退，反而在大踏步前进！

　　五年来辉煌成就的取得，是在党中央、省委、省政府和市委的正确领导下，全市广大干部群众同心同德、艰苦奋斗的结果，是720万沈阳人民辛勤汗水和聪明才智的结晶。在这里，我代表市政府，向投身于沈阳改革开放和现代化建设的各族人民致以亲切的问候和崇高的敬意！向给予我们支持与监督的人大代表、政协委员，各民主党派、工商联和无党派人士、人民团体，向参与沈阳发展建设的中央和省在沈单位、驻沈部队指战员、武警官兵和在沈工作的外籍人士、港澳台同胞、海外侨胞及国际友人表示衷心的感谢！

　　总结过去的五年，我们清醒地看到政府工作还存在许多问题。沈阳的综合实力在大城市中的位次后移，中心城市功能发挥不够；工业支柱产业不强，企业规模不大；社会保障工作压力较大，城镇和农村就业矛盾突出；人民群众生活，尤其是低收入家庭生活还存在不少困难，社会中还存在着种种不稳定因素；部分机关干部的思想观念、工作作风和工作方法还不适应新形势的要求，官僚主义和形式主义还不同程度地存在。对于以上问题，我们将在今后的工作中认真加以解决。二、今后五年的奋斗目标和主要任务

　　未来的五年，是沈阳经济与社会快速发展的重要战略机遇期。党的十六大提出的全面建成小康社会的宏伟目标，对我们加快经济和社会发展提出了新的要求，业已形成的诸多极为有利的内、外部条件，使我们谋求快速发展成为可能。

　　世界范围迅猛发展的经济结构调整，为我市承接发达国家产业转移，提供了难得的机遇；南方资本加速向北拓展，为我市经济的成长壮大创造了良好的商业机会；老工业基地改造和工业化进程的深化，形成了巨大的产业投资需求；众多的周边城市和幅员辽阔的腹地，创造了少有的巨大市场；市级五大开发区和县区工业园的全面启动，为外来投资者提供了广阔的发展空间；规模巨大的国有资产存量和经验丰富的产业职工大军，为我们准备了丰裕的生产要素；经济体制改革的不断深化和日臻完善的城市环境，大幅度提高了中外客商对沈阳发展的预期；“制博会”的成功举办和先进装备制造基地试点落户沈阳，开启了我市现代装备制造业加速发展的大门；一大批骨干企业和重大项目的健康培育，正在为沈阳的经济腾飞构筑起强大的支撑。所有这一切，都将会推进沈阳经济进入新一轮的高速发展期。

　　更为重要的是，经过长期的实践和探索，全市已经清晰地形成了利用重要战略机遇期实现跨越式发展，全面建成小康社会的思路，重振沈阳雄风已经成为720万人民群众坚定不移的信念。

　　只要我们万众一心，团结奋斗，就一定能够把沈阳建设成投资创业的热土、美好生活的乐园！

　　今后五年政府工作的指导思想是：

　　认真贯彻落实党的十六大精神，高举邓小平理论伟大旗帜，努力实践“三个代表”重要思想，以市委十届六次全会关于全面建成小康社会的要求为目标，以发展为主题，以结构调整为主线，以改革开放和科技进步为动力，以提高人民生活水平为根本出发点，坚持工业立市，实施开放牵动、科教兴市、中心城市功能拓展和可持续发展战略，在进一步巩固和发展国有经济的同时，大力发展非公有制经济，全面增强城市的综合竞争力，努力把沈阳建设成区域性的商贸、金融、信息中心和先进装备制造、高新技术产业化、农业产业化基地，实现全市经济的跨越式发展和社会全面进步。

　　今后五年全市经济和社会发展的奋斗目标是：

　　——国民经济发展再上新台阶，主要经济指标基本实现翻一番。用五年或者更长一点时间，全市国内生产总值要突破2500亿元，固定资产投资突破800亿元，财政收入突破200亿元，外商直接投资突破30亿美元，争取各项主要经济指标在东北地区达到领先水平，为称雄中国北方打下基础。

　　——全面深化农业和农村经济结构调整，实现传统农业向现代农业的初步转变。要采取切实措施解决“三农”问题，促进农民增收致富。农村经济结构调整要取得新进展，二、三产业占县区经济总量的比重要达到90％以上，粮经作物比例达到5：5。农村城市化水平达到40％，提高11个百分点。农业高新技术开发区跻身全国先进农业开发区前列。

　　——全面完成国有经济战略性调整任务，建立起布局合理、结构优化的现代工业体系。具备条件的大型国有企业，要经过整体合资进行公司制改造，国有中小企业全部放开，问题企业全部解决。

　　积极发展汽车、装备制造、电子信息、医药化工和食品饮料等产业，形成综合配套齐全、核心竞争力强劲、市场规模持续扩大的支柱产业群。汽车及零部件工业产值突破800亿元，装备制造业产值突破400亿元。

　　完成100户产值超10亿元的骨干企业群和100个重大项目的培育任务，新增工业产值1000亿元。浑南新区全面开发任务大部分完成。铁西新区初步建成国内一流的先进装备制造基地。要以信息化带动工业化，走新型工业化道路。

　　今后五年，全市工业总产值和工业增加值要分别保持15％和13％以上的增长速度。

　　——实现森林城市的建设目标，城市基础设施、水系和生态环境建设水平显著提高。到2007年，城区绿化覆盖率达到40％以上，人均公共绿地面积达到12平方米，城区水面积率达到4.5％，基本建成森林城市；现代交通组织体系初步形成，每万人拥有公交车达到15台；农村居住条件和出行条件进一步改善，实现村村通柏油路；城市垃圾处理率达到100％，污水处理率达到85％，全年大气优良天数达到300天，全面完成环保模范城市的创建任务，生态环境质量达到全国重点环保城市先进水平。要经过五年的努力，全部解决棚户区问题。

　　——全面提高对外开放水平，经济外向度和城市国际化水平显著提高。充分利用入世的机会，形成全方位、多层次、宽领域的对外开放格局，其中特别要做好对韩国、日本的开放招商工作。努力使沈阳成为外商投资不断增加、国际航线不断增开、常住外国人口不断增长、来访外国政要不断增多的国际化大都市。

　　——建立完善的社会主义市场经济体制，市场在资源配置中的基础性作用充分发挥。全面建立起比较完备的投融资体系、国有资产经营和监督体系、城市建设投融资体系、商品和生产要素市场体系、中介服务体系和信用体系，形成开放、竞争、有序的经济管理体制。

　　——大力发展现代服务业，积极拓展中心城市功能。深化流通体制改革，优化服务业结构，加快改造传统服务业步伐，集中发展重点商业街区、新型商业业态和现代物流配送体系，努力提高服务业的整体素质和国际竞争力。

　　——各项社会事业全面发展，市民整体素质进一步提高。逐步建立起以为人民服务为核心，以集体主义为原则，以诚实守信为重点的社会主义思想道德体系，市民的道德素质、科学文化素质和健康素质明显提高；在进一步完善功能设施的基础上，形成比较完备的现代国民教育体系、科学和文化创新体系、全民健身和医疗卫生体系；深入开展城乡共建精神文明活动，通过建设高标准文明村活动，使村镇环境面貌明显改善；初步建成现代意识浓郁、社会行为规范、文化生活丰富、全国一流的文化名城。

　　——城乡人民收入明显提高，人民生活整体水平迈向比较富裕阶段。城市居民人均可支配收入达到1.1万元、农民人均纯收入达到5400元，城乡居民生活更加富足、殷实；人居环境明显改善，城市人均住房建筑面积达到26平方米，农村居住条件进一步改善。继续完善社会保障体系，就业和再就业压力明显减轻，争取步入良性循环，并跻身于副省级城市先进行列。

　　我们要经过五年的艰苦努力，把沈阳初步建设成以现代工业、现代服务业和现代农业为支撑，社会保障体系完善，文化生活丰富多彩，经济生活规则化、政治生活民主化、社会生活法治化，环境优美、生活富庶、人气旺盛，在世界具有一定影响力的国际化大都市。

　　沈阳的未来将十分美好，我们应该为自己的城市感到自豪，更应该为自己城市的发展贡献出一切力量！三、2003年的主要工作安排

　　2003年全市经济和社会发展的主要指标预期为：国内生产总值增长11％；财政收入增长13％；全社会固定资产投资增长12％；实际利用外资增长10％；社会消费品零售总额增长11％；城市居民可支配收入和农民纯收入分别增长8％和6％。

　　（一）以开展项目年活动为依托，积极扩大对内对外开放。市委、市政府将今年确定为项目年，其目的是在全市上下形成一种氛围，集中广大干部群众的智慧和力量，通过建设一批大项目、好项目，全面推动经济和社会事业的快速发展。此举对于增强沈阳的发展后劲，具有决定性意义。

　　项目年的工作目标是，力争实现利用外资20亿美元；从国内引进1000户规模以上工商企业；开工建设并竣工一批项目。

　　为了全面落实项目年活动，针对不同地区、不同行业下达项目任务，并作为领导干部业绩考核的主要指标，要采取一系列有效方法和措施，务求活动取得实质性成果。

　　（二）以大规模发展林业为突破口，积极调整农业结构。退耕还林、植树造林，不仅有生态效益，更有经济效益。农村植树造林面积要突破100万亩。要加快发展林产品深加工企业。

　　要加快农业产业化步伐，大力发展龙头企业，重点扶持种子、蔬菜等十大产业链建设，加速农业现代园区的发展。要通过发展养殖业，带动农村产业结构调整。要大力加强农村社会化服务体系和科技创新体系的建设。要认真开展农产品的标准化工作，推进无公害生产。要进一步开拓国内外市场，积极建设针对韩、日、俄等国的蔬菜和花卉生产基地。沈阳农业高新区建设要取得更大的进展。

　　县城和副城建设要迈出新的步伐，市政府将继续给予县城和副城建设各1000万元的资金支持。

　　（三）以重振工业重镇雄风为目标，完成国有企业战略重组任务。国有企业改革已进行多年，今年我们要全面完成战略重组任务。20户大企业整体合资年内务必完成，各类国有中小企业年内全部放开搞活，各种问题企业年内基本处理完毕。为了实现上述目标，要在财政继续支付改革成本的同时，加大国有企业职工并轨和社会保障的工作力度。

　　要大力发展汽车及零部件、装备制造、电子信息、医药化工和食品饮料等支柱产业。要积极推进建设100户产值超10亿元的企业和100个重大项目，形成实力雄厚的骨干工业企业群。要重点扶持那些高效益、高纳税和高就业的企业。

　　浑南新区要以绿化为主继续改善投资环境，全年实际利用外资要达到4亿美元。要全面启动建设科峰芯片等100户重点企业。

　　铁西新区要通过创造环境，利用新区在发展空间、融资手段和级差地租等方面的优势，引进外资4亿美元，实现50户企业搬迁和重组，引进100户企业在区内落户。

　　各区、县（市）要参照浑南新区和铁西新区的模式，借鉴国内外先进园区建设经验，加快工业园区建设，不仅要出形象，还要出进度，工业总产值年均递增速度要高于50％。

　　（四）以发展现代服务业为载体，进一步拓展中心城市功能。全市商贸流通业要适应新形势要求，加快建设和发展沈河中央商务区，集中改造和建设太原街、中街、长江街、北市和西塔街等五大繁华商业街区，使其达到全国领先水平。大力发展新型商业业态和社区服务业，积极推进连锁经营，加速商品市场的转型升级，发展现代物流配送，吸引更多的国内外大型流通企业在我市设立总部和采购、配送中心。

　　要充分发挥会展经济、旅游经济的综合拉动作用。要努力争办国家级和国际性的大型展会，进一步办好“制博会”。棋盘山风景旅游区要在高标准规划前提下，全面启动基础设施建设，抓紧引进一批大项目。要充分利用保护性建筑周边环境整治和城市建设的新成果，进一步发展旅游业。

　　要积极发展和壮大金融业。要吸引国内外金融机构在我市设立分支机构或代表处，积极引进和设立风险投资公司，使全市风险投资规模超过20亿元。同时，还要进一步加大各类市场和中介机构的发展力度，发展电子商务等信息服务业。

　　（五）以努力改善发展环境为前提，促进民营经济的快速发展。要坚持“放心、放开、放手”的方针，努力促进民营经济快速发展。

　　各区、县（市）要把发展民营经济作为推进当地经济发展的主要途径，要采取各种积极方式，以县区工业园区为主要载体，引进外地工商企业，建立“企业家苗圃”，壮大企业家队伍，活跃中介服务，消除各种阻碍因素，促进民营经济上规模、上档次、上水平。

　　要进一步认真落实《关于加快发展民营经济的若干意见》，在放宽市场准入、政府审批服务、完善法律服务体系、拓宽融资渠道、加强境外上市，以及传递信息、信用担保、人才培训等方面，给予民营经济实实在在的支持。

　　（六）以绿化、道路和水系建设为重点，加快城市建设步伐。今年，城区要新增绿地17平方公里，加快二、三环之间40平方公里的经济林和生态林的建设步伐。年内实现绿地率31％、绿化覆盖率35％和人均公共绿地8平方米。

　　要整修道路300万平方米，再建设10条景观路。争取国家早日批准地铁工程项目并开工建设。

　　要按照产业化原则，在将集中供热率提高到70％的基础上，今年再拆除1000根烟囱和400座锅炉房；加大煤烟治理、尾气控制和扬尘整治力度，确保大气优良天数提高到240天。

　　要以浑河为重点抓好水系建设，今年要集中建设和整治浑河青年桥至工农桥5.7公里段的水面和绿地。

　　要新增公交车1000台，新开辟公交线路20条，延伸、调整线路10条，更新线路10条，村通柏油路1000公里。继续做好保护性建筑周边整治及内部整修工作。

　　要整治住宅小区环境，推进拆违建绿。选择一部分住宅进行改造，提升居住水平。要在农村开展村屯环境整治工作。

　　加强城市建设的关键是筹措资金，今年土地出让金征收要在去年实现15亿元的基础上，达到20亿元，用于城市建设。

　　（七）以推进再就业工程为重点，进一步完善社会保障体系。进一步做好养老保险费和失业保险费的征收工作，养老保险费征收要达到22亿元，失业保险费征收要达到2.8亿元；要继续扩大基本医疗保险覆盖面，参保人数要达到150万人；要坚持做好和不断完善城市最低生活保障工作，继续做到应保尽保；年内要实现10万名国有企业职工并轨。

　　要不断完善再就业扶持政策，不断改善创业环境，创造就业机会、增加就业岗位，充分调动各种积极因素，进一步做好就业、再就业工作。今年要开发就业岗位12万个，实现再就业18.5万人次。

　　农村扶贫工作要从救济型向开发型转变，重点开发100个村，确保10万贫困人口解决温饱问题，5万人脱贫致富。

　　（八）以大文化建设为中心，全面推进社会事业发展。要深入推进科研机构的市场化、产业化进程，促进科研成果向现实生产力转化；认真开展“用高新技术改造传统装备制造业基地建设”的试点工作，抓好重大科技专项和高新技术产业化重点项目；要努力完善技术创新体系、创业孵化体系、风险投资体系和技术产权交易市场体系。认真实施“人才高地”计划，积极引进和培养高层次紧缺人才，吸引更多的海外留学人员回国创业。

　　教育事业要以深化教育综合改革为突破口，努力办好各级各类教育，提高办学质量。对非义务教育，提倡投资主体多元化办学。要十分重视发挥在沈的国家、省属院校的积极作用，通过办好这些学校，进一步提升沈阳的城市品位。

　　加快文化体制改革和机制创新工作。实施品牌工程和精品工程，办好第五届沈阳艺术节，推出一批文化精品。进一步活跃城乡群众文化生活。积极发展文化产业，开发文博资源，抓好重点文化产业项目的招商引资。做好“一宫两陵”申报世界文化遗产的迎检工作。建设具有鲜明时代精神、民族风格和沈阳特色的现代都市文化。

　　积极推进农村卫生和城镇医疗卫生体制改革。年内完成全市的卫生监督和疾病控制体制改革工作。进一步完善医疗服务体系、公共卫生服务体系和卫生执法体系，提高医疗服务质量。

　　加大体育市场培育和开发力度，切实抓好体育事业单位的体制改革试点。以实施《全民健身计划纲要》第二期工程为主线，广泛开展全民健身活动，提高市民身体素质和竞技体育水平。

　　计划生育、统计、审计、物价、民族、宗教、参事、文史、广播、电视、新闻、出版、档案、修志、老龄、残联、民兵、预备役、双拥、人防、侨务、对台、防震、气象等项工作也要取得新的进展。

　　（九）以全面推进“三化”为指针，推进民主法制的进程。按照经济生活规则化、政治生活民主化、社会生活法治化的要求，积极推进民主决策、民主管理和民主监督，探索和建立一整套深入了解民情、充分反映民意、广泛集中民智、切实珍惜民力的决策机制。

　　要自觉接受人大的法律监督和政协的民主监督，要尊重人大代表和政协委员的权利，认真办理好人大代表和政协委员的议案、提案和建议。要完善职工代表大会制度和村民自治、居民自治等制度，培养和提高公民参与民主管理的意识、能力和水平。

　　要大力推进依法治市，推进政府决策的科学化、民主化和法制化。要切实加强依法行政，全面实施行政执法责任制和评议考核制。要完善监督制度，切实加强干部队伍作风建设，深入开展理论与业务的学习活动，努力提高干部队伍的整体素质。要转变政府职能，继续深化审批制度改革。要进一步推进政务公开。要继续深入开展反腐败斗争，提高政府机关建设水平。

　　要全力维护社会稳定，坚持“打防结合，预防为主”的方针，严厉打击各种严重刑事犯罪和经济犯罪。要建立和完善各级信访、市民投诉和社区人民调解机制，扎实、有效地化解人民内部矛盾，积极稳妥地处置群体性事件，切实增强社会治安的防控能力。

　　信用是健全的市场经济体制的基石，没有信用观念和完备的信用体系，就不可能保证经济的健康发展。今年，全市要集中相当精力进行信用体系建设，要按照信用规则的要求，全面启动征信系统、信用评价系统、信用查询系统、信用服务系统和信用管理系统的建设，努力培育信用企业、信用个人和信用政府。

　　各位代表、同志们！

　　新的进军号角已经吹响，我们踏上了全面建成小康社会的新征程。尽管前进的道路上还有许多艰难险阻，但我们对未来充满了必胜的信心！有党的十六大精神的指引，有以胡锦涛为总书记的党中央，以及省委、省政府和市委的正确领导，有市人大的法律监督和市政协的民主监督，有720万沈阳人民的努力拼搏，沈阳一定能重振雄风，沈阳的明天一定会更美好！
